# Even low level of physical activity is associated with reduced mortality among people with metabolic syndrome, a population based study (the HUNT 2 study, Norway)

**DOI:** 10.1186/1741-7015-9-109

**Published:** 2011-09-29

**Authors:** Dorthe Stensvold, Javaid Nauman, Tom IL Nilsen, Ulrik Wisløff, Stig A Slørdahl, Lars Vatten

**Affiliations:** 1 K.G. Jebsen Center of Exercise in Medicine, Department of Circulation and Medical Imaging, Norwegian University of Science and Technology, Trondheim, Norway; 2 Department of Circulation and Medical Imaging, Norwegian University of Science and Technology, Trondheim, Norway; 3 St. Olavs Hospital, Postboks 3250 Sluppen, 7006 Trondheim, Norway; 4 Human Movement Science Programme, Norwegian University of Science and Technology, Dragvoll, Loholt allé 81, Trondheim, Norway; 5 Department of Public Health, Norwegian University of Science and Technology, Postboks 8905, MTFS, 7491 Trondheim, Norway

**Keywords:** metabolic syndrome, physical activity, mortality

## Abstract

**Background:**

Low levels of physical activity may increase the risk of developing metabolic syndrome, a cluster of metabolic factors that are associated with the risk of premature death. It has been suggested that physical activity may reduce the impact of factors associated with metabolic syndrome, but it is not known whether physical activity may reduce mortality in people with metabolic syndrome.

**Methods:**

In a prospective study of 50,339 people, 13,449 had metabolic syndrome at baseline and were followed up for ten years to assess cause-specific mortality. The population was divided into two age groups: those younger than 65 years of age and those older than age 65. Information on their physical activity levels was collected at baseline.

**Results:**

Metabolic syndrome was associated with higher mortality from all causes (hazard ratio (HR) 1.35, 95% confidence interval (95% CI) 1.20 to 1.52) and from cardiovascular causes (HR 1.78, 95% CI 1.39 to 2.29) in people younger than 65 years old than among other populations. In older people, there was no overall association of metabolic syndrome with mortality. People with metabolic syndrome who reported high levels of physical activity at baseline were at a reduced risk of death from all causes compared to those who reported no physical activity, both in the younger age group (HR 0.52, 95% CI 0.37 to 0.73) and in the older age group (HR 0.59, 95% CI 0.47 to 0.74).

**Conclusion:**

Among people with metabolic syndrome, physical activity was associated with reduced mortality from all causes and from cardiovascular causes. Compared to inactivity, even low levels of physical activity were associated with reduced mortality.

## Background

Metabolic syndrome, a clustering of metabolic risk factors, increases the risk of premature death from cardiovascular disease (CVD) threefold compared to people without metabolic syndrome [1]. In the Western world, approximately 25% of people in the 40- to 49-year-old age group and approximately 45% in the 60- to 69-year-old age group may have metabolic syndrome, but its prevalence appears to differ between populations and is dependent on age, gender and ethnicity [2,3].

There is no single medication available to treat metabolic syndrome, and each clustering factor needs to be treated separately [4]. Lifestyle changes are highly recommended as part of the treatment strategy [5,6]. The results of population-based studies have suggested that a low level of physical activity is associated with a high prevalence of metabolic syndrome [7-10], and clinical studies have shown that exercise training may improve the risk factor profile for people with established metabolic syndrome [11-13]. According to current recommendations, people should engage in 30 minutes of moderately intense activity five days per week or vigorous aerobic physical activity for a minimum of 20 minutes three days per week to promote and maintain good health [14,15]. Alternatively, people can engage in a combination of moderate and vigorous activity to meet the recommended guidelines. In addition, activities that maintain or increase muscular strength and endurance are recommended for a minimum of two days each week. It is not known whether people with metabolic syndrome will benefit from a similar level of physical activity, and it is also not known whether physical activity may reduce mortality in people with metabolic syndrome.

In a prospective study of more than 13,000 men and women with metabolic syndrome, we assessed whether self-reported levels of physical activity are associated with the risk of death from all causes and specifically with the risk of death from cardiovascular causes.

## Methods

### Study population

An adult population ages 20 years and older in Nord-Trøndelag County in Norway was invited to participate in a large health survey (the Nord-Trøndelag Health Study 2, or HUNT 2) from 1995 to 1997. Of 92,205 individuals, 65,215 (70.7%) accepted the invitation to enter the study and underwent a medical examination that has been described in detail elsewhere [16]. Briefly, the participants filled in self-administered questionnaires and were subjected to physical measurements, including anthropometric factors and blood pressure, and a blood sample was taken from all participants. Among many health-related items, the questionnaire covered physical activity, smoking habits, alcohol consumption, marital status, occupation and educational attainment.

### Exclusion criteria

On the basis of self-reports, we initially excluded 5,220 participants from mortality follow-up if they had a history of stroke or myocardial infarction or known angina pectoris. Important information regarding physical activity was missing for 9,656 people, and these initial participants were also excluded. Therefore, the present study included 50,339 participants (13,449 with metabolic syndrome and 36,890 without metabolic syndrome) who were followed up for cause-specific mortality.

### End points and follow-up

Compulsory reporting of deaths by public health officers and physicians is the basis for the Causes of Death Registry in Norway, including coding of underlying causes of death. End points in this study were deaths from any cause and deaths from cardiovascular causes (International Classification of Disease (ICD)-9:390 to 9:459; ICD-10:I00 to 10:I 99).

### Serum glucose levels

Since blood samples were drawn throughout the day and fasting samples could not be obtained, the time since the most recent meal was carefully recorded. In the analyses, it was adjusted for the time since the most recent meal to compensate for the nonfasting state of the participants. A total of 10,420 individuals (16%) reported that at least four hours had passed since their most recent meal, and their glucose levels were used as one factor in the identification of people with prevalent metabolic syndrome.

### Metabolic syndrome

Metabolic syndrome was defined as the concurrent prevalence of any three of the following five factors [17]: waist circumference ≥94 cm for men and ≥80 cm for women, serum triglyceride level ≥1.7 mmol/L, high-density lipoprotein < 1.0 mmol/L in males and < 1.3 mmol/L in females, systolic blood pressure ≥130 mmHg or diastolic blood pressure ≥85 mm Hg or treatment for hypertension, fasting plasma glucose level ≥5.6 mmol/L, or previously diagnosed or known type 2 diabetes.

### Physical activity

The participants were asked about their physical activity (PA) during leisure time. The PA questions differentiated between light and hard physical activity during an ordinary week, with four response options for each intensity level (none, less than one hour, one to two hours or at least three hours). The questions about hard and light PA were not mutually exclusive, and the data allowed us to construct a PA variable that roughly corresponded to the current recommendations for aerobic physical activity [14]. Moderate intensity PA has previously been described as being equivalent to brisk walking [14], and we therefore assumed that this response would correspond to what people reported as light PA. In the constructed PA variable, duration of exercise was divided into light PA (less than one hour and at least three hours of light activity) and hard PA (less than one hour and at least one hour of hard activity). Furthermore, we combined hard and light PA into the following four activity categories: Inactive (inactive regarding both light and hard PA), Low (less than three hours of light PA and no hard PA), Moderate (less than three hours of light PA and less than one hour of hard PA or at least three hours of light PA and no hard PA) and High (at least three hours of light PA and less than one hour of hard PA, at least three hours of light PA and at least one hour of high PA, or less than three hours of light PA and at least one hour of hard PA). Thus, low PA included both those who reported less than one hour of light PA per week and those who reported up to two hours of light PA per week. Low PA indicated a PA level lower than what is recommended. Moderate PA corresponded fairly well with the current PA recommendations as it included three hours or more of light PA or less than three hours of light PA and less than one hour of hard PA (including moderate and/or vigorous PA). High PA indicated an activity level higher than the minimum recommended level.

### Statistical analysis

Person-years were calculated from the date of entrance into the study until the date of death or until the end of follow-up, 31 December 2007, whichever occurred first. Cox regression analyses were used to compute hazard ratios (HRs) of all-cause and cardiovascular mortality, where the rate of death of people with metabolic syndrome was compared with the mortality rate in people without metabolic syndrome. The precision of the estimates was assessed on the basis of 95% confidence intervals (95% CI). In similar analyses, we estimated the association of PA with the risk of death among people with metabolic syndrome using inactive participants as the reference category. All estimated associations were adjusted for age by including current age as the time scale in the Cox regression model. In additional multivariable models, we adjusted for the potential confounding effects of sex, marital status (married, unmarried, divorced or separated and widow or widower), education level attained (≤10 years, 11 to 14 years, ≥15 years or unknown), alcohol consumption (frequency during the past two weeks: none, one to four, at least five, total abstainer or unknown), smoking status (never, former, current or unknown) and occupational activity (mostly sedentary, much walking, much walking and lifting, heavy physical work or unknown).

In a separate analysis, we examined the combined effect of PA and metabolic syndrome status on the risk of death from all causes and specifically from CVD. For this purpose, participants who were without metabolic syndrome and reported high PA served as the reference group. Departure from the proportional hazards assumption was evaluated by Schoenfeld residuals, and, in the analysis, we found no evidence of departure. Potential effect modification was assessed for age at baseline and sex. *P *values for interaction were calculated by using likelihood ratio tests to compare models with and without the interaction terms. *P *values for linear trends were calculated by using PA categories as an ordinal variable in the Cox regression model. All statistical tests were two-sided, and the statistical analyses were conducted using Stata for Windows version 10.0 software (StataCorp LP, Texas 77845, USA).

### Ethics

All participants in the HUNT 2 study provided their written consent, and the Regional Committee for Medical Research Ethics approved the study.

## Results

### Interaction

There was a significant interaction with age, but not with sex. Therefore, the study population was divided into two age groups (ages < 65 years and ≥65 years), and data for men and women were combined.

### Baseline characteristics

Baseline characteristics of the populations are presented in Table 1. The total prevalence of metabolic syndrome was 27%. Its prevalence at baseline was 23% among participants younger than 65 years of age and 44% among participants 65 years or older. In the younger age group, 53% reported a level of exercise that was equal to or higher than current recommendations, and only 9% reported that they were inactive (Table 1). In the older age group, the corresponding proportions were 41% and 19% (Table 1).

**Table 1:** Descriptive statistics of people with metabolic syndrome in HUNT 2^a^

	Age group, years	
	
Demographics	< 65	≥65
Participants, *n*	9,883	3,566
Person-years,	124,466	50,269
Deaths (all causes), *n*	460	1,379
Deaths (CVD), *n *(% of all deaths)	119 (26)	592 (43)
Mean age at baseline, years (± SD)	45.8 (11.3)	73.1 (6.1)
Mean body mass index, kg/m^2 ^(± SD)	29.5 (4.0)	29.0 (3.9)
Mean waist circumference, cm (± SD)	95.3 (10.0)	95.1 (10.3)
Mean SBP, mmHg (± SD)	142.5 (17.3)	160.9 (22.3)
Mean DBP, mmHg (± SD)	85.1 (11.0)	87.7 (12.9)
Mean serum triglycerides, mmol/L (± SD)	2.8 (1.4)	2.6 (1.1)
Mean serum glucose, mmol/L (± SD)	5.6 (1.7)	6.3 (2.4)
Mean serum HDL, mmol/L (± SD)	1.1 (0.3)	1.2 (0.3)
Mean percentage of people taking diabetes medication (± SD)	3.8 (0.04)	12.6 (0.1)
Mean percentage of people taking BP medication (± SD)	11.5 (0.1)	33 (0.3)
Mean percentage of current smokers (± SD)	32.2 (0.3)	15.9 (0.1)
Mean percentage of sedentary occupational work (± SD)	28 (0.2)	45 (0.3)
Mean percentage of college or university education (± SD)	18 (0.2)	5 (0.05)
Participants' PA levels^b^, %		
Inactive	9	19
Low	38	39
Moderate	27	29
High	26	12

^a^HUNT 2, Nord-Trøndelag Health Study 2; CVD, cardiovascular disease; SBP, systolic blood pressure; DBP, diastolic blood pressure; HDL, high-density lipoprotein; PA, physical activity; Inactive, no light and no hard physical activity; Low, less than three hours of light and no hard PA; Medium, less than three hours light and less than one hour hard PA, or at least three hours light PA and no hard PA; High, at least three hours light PA and less than one hour hard PA or at least three hours light PA and at least one hour hard PA or less than three hours light PA and at least one hour hard PA; ^b^because of rounding, percentages may not total 100.

### Metabolic syndrome and mortality

During a mean follow-up period of 10 years (standard deviation ± 2 years), 1,839 persons among 13,449 with metabolic syndrome died. In the group younger than 65 years of age, 26% of the deaths were caused by CVD, and among people age 65 years or older, 43% of the deaths were caused by CVD.

**Figure 1:**
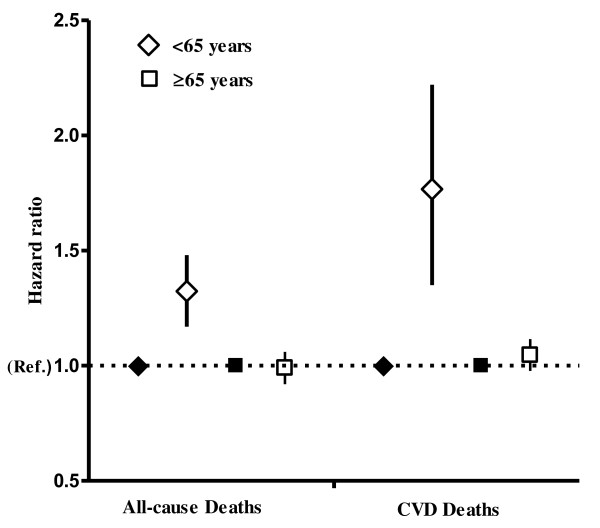
**Hazard ratios of all-cause and cardiovascular disease-related mortality among people with metabolic syndrome. **Adjusted for age (continuous), sex (men and women), smoking status (never, former, current or unknown), physical activity index (High, Medium, Low or Inactive), marital status (married or unmarried, widow or widower, divorced or separated), education level attained (≤10 years, 11 to 14 years, ≥15 years or unknown), alcohol consumption (frequency during past two weeks: none, one to four, at least five or total abstainer) and occupational activity (mostly sedentary, much walking, much walking and lifting, heavy physical work or unknown).Squares and diamonds represent risk estimates, and bars represent 95% CI. People without metabolic syndrome (represented by coloured diamonds and squares) served as the reference group.

Among people younger than 65 years of age, those with metabolic syndrome were at higher risk of death from all causes (HR 1.35, 95% CI 1.20 to 1.52) and from CVD (HR 1.78, 95% CI 1.39 to 2.29) compared to people without metabolic syndrome. In the older age group, there was no clear association of metabolic syndrome with the risk of dying (Figure 1).

### Physical activity and mortality

Table 2 shows the relative risk of dying from all causes and from CVD among people with metabolic syndrome. The table shows that after adjustment for potentially confounding factors, the risk of death from all causes was reduced with increasing PA level (*P *trend < 0.001). Highly active people younger than 65 years of age had a strong risk reduction in all-cause mortality (HR 0.52, 95% CI 0.37 to 0.73) compared to inactive people. In the older age group (≥65 years), the corresponding comparison showed a similar risk reduction (HR 0.59, 95% CI 0.47 to 0.74).

**Table 2:** Levels of physical activity in people with metabolic syndrome and HRs of all-cause and CVD-related deaths^a^

	All-cause mortality						CVD-related deaths			
	
PA index	Number	Person-years	Deaths	HR^b^	HR^c ^(95% CI)	*P *trend	Deaths	HR^b^	HR^c ^(95% CI)	*P *trend
< 65 years old	9,883									
Inactive^c^		9,357	70	1.0	1.0 (Ref.)		15	1.0	1.0 (reference value)	
Low		41,723	203	0.62	0.71 (0.54 to 0.94)		61	0.87	1.03 (0.58 to 1.83)	
Moderate		30,398	114	0.52	0.58 (0.43 to 0.79)		26	0.56	0.63 (0.33 to 1.20)	
High		28,119	73	0.46	0.52 (0.37 to 0.73)		17	0.51	0.60 (0.29 to 1.22)	
						< 0.001				0.02
≥65 years old	3,566									
Inactive^d^		5,620	375	1.0	1.0 (Ref.)		171	1.0	1.0 (reference value)	
Low		13,347	522	0.76	0.75 (0.65 to 0.86)		235	0.76	0.76 (0.62 to 0.93)	
Moderate		10,157	361	0.70	0.65 (0.56 to 0.76)		140	0.61	0.58 (0.46 to 0.74)	
High		4,293	121	0.69	0.59 (0.47 to 0.74)		46	0.59	0.52 (0.37 to 0.73)	
						< 0.001				< 0.001

^a^CVD, cardiovascular disease; PA, physical activity; HR, hazard ratio; CI, confidence interval; ^b^adjusted for age (continuous); ^c^adjusted for age (continuous), sex (men and women), marital status (married or unmarried, widow or widower, divorced or separated), education level attained (≤10 years, 11 to 14 years, ≥15 years and unknown), alcohol consumption (frequency during past two weeks: none, one to four, at least five, total abstainer and unknown), smoking status (never, former, current or unknown), occupational activity (mostly sedentary, much walking, much walking and lifting or heavy physical work); ^d^inactive participants served as the reference group.

The results related to deaths from CVD were similar to those for all-cause mortality. Thus, the reduction in the risk of dying from CVD related to increasing PA level showed a linear trend (*P *trend = 0.02 for people < 65 years of age and *P *< 0.001 for people ≥65 years of age), and the HRs for highly active people was 0.60 (95% CI: 0.29 to 1.22) for people younger than 65 years old and 0.52 (95% CI: 0.37 to 0.73) for people age 65 years or older.

### Metabolic syndrome status and physical activity

We also assessed the combined effects of PA and metabolic syndrome in the total population younger than 65 years of age (Table 3). Our data showed that the association between PA and mortality displayed the same pattern in people with and without metabolic syndrome. Interestingly, people with metabolic syndrome who reported high levels of PA had only a slightly higher mortality risk compared to their healthy counterparts (adjusted HR: 1.13, 95% CI: 0.87 to 1.49). In older people with metabolic syndrome, the risk for premature death was not significantly higher compared to older people without metabolic syndrome. Thus, the impact of PA was the same in people with and people without metabolic syndrome ≥65 years old (data not shown).

**Table 3:** Adjusted HRs of all-cause and CVD-related deaths related to physical activity and MetS status among people younger than 65 years old^a^

	Physical activity index			
	
Mortality	High	Moderate	Low	Inactive
Total deaths				
Without MetS				
Deaths	193	200	265	83
Person-years	134,119	99,214	112,980	22,046
HR (95% CI)	1.0 (reference value)^b^	1.02 (0.84 to 1.25)	1.08 (0.89 to 1.30)	1.54 (1.18 to 2.00)
				
With MetS				
Deaths	73	114	203	70
Person-years	28,119	30,399	41,723	9,357
HR (95% CI)	1.13 (0.87 to 1.49)	1.26 (0.99 to 1.59)	1.56 (1.28 to 1.92)	2.13 (1.61 to 2.83)
				
CVD-related deaths				
Without MetS				
Deaths	31	41	48	17
Person-years	134,119	99,214	112,980	22,046
HR (95% CI)	1.0 (reference value)^b^	1.29 (0.81 to 2.07)	1.18 (0.74 to 1.87)	1.76 (0.97 to 3.22)
				
With MetS				
Deaths	17	26	61	15
Person-years	28,119	30,399	41,723	9,357
HR (95% CI)	1.51 (0.83 to 2.73)	1.63 (0.96 to 2.77)	2.75 (1.76 to 4.30)	2.55 (1.35 to 4.79)

^a^MetS, metabolic syndrome; HR, hazard ratio; CI, confidence interval; MetS, metabolic syndrome; CVD, cardiovascular disease; ^b^participants who reported high physical activity levels and did not have metabolic syndrome served as the reference group. HRs are adjusted for age (continuous), sex (men and women), marital status (married or unmarried, widow or widower, divorced or separated), education level attained (≤10 years, 11 to 14 years, ≥15 years or unknown), alcohol consumption (frequency during past two weeks: none, one to four, at least five, total abstainer or unknown), smoking status (never, former, current or unknown), occupational activity (mostly sedentary, much walking, much walking and lifting or heavy physical work).

## Discussion

In this large, prospective population study of men and women with metabolic syndrome, we found a gradual reduction in all-cause mortality associated with increasing levels of PA. Importantly, the greatest mortality difference was found between people who reported no activity and those who reported low levels of PA.

To our knowledge, this is the first study to evaluate the association of PA with mortality among people with metabolic syndrome. The main finding was in line with prospective studies in the general population [18-21] in showing that PA is associated with a reduction in mortality from all causes, and specifically from CVD, among people with metabolic syndrome. The substantial mortality difference that we found between low levels of PA and no activity also corresponds to findings in the general population [21-23]. Less than 50% of adults in the US meet the recommended level of PA [22], but our results suggest that even PA at a level that most people are able to achieve is likely to reduce mortality in people with metabolic syndrome.

We cannot exclude the possibility that unknown factors other than PA might explain the high mortality related to physical inactivity. It is possible that being inactive reflects prevalent illness and that therefore the stronger association with mortality might be as expected. We attempted to take this possibility into account by excluding participants with a history of stroke, myocardial infarction or known angina pectoris.

We found no difference regarding risk in mortality due to CVD between those who reported being inactive and those who reported low levels of PA among people with metabolic syndrome in the age group younger than 65 years of age. However, that analysis was based on a small number of deaths, and thus the statistical power required to detect any effect was modest.

A number of studies have shown that metabolic syndrome is associated with increased risk of death from all causes, and specifically from CVD, compared to people without metabolic syndrome [1,5]. In our study, there was a positive association of metabolic syndrome with all-cause and CVD mortality only among people younger than 65 years of age. Our finding is in line with that of Hildrum *et al*. [24], who reported higher all-cause mortality associated with metabolic syndrome in middle-aged people but not in older adults. A possible explanation for our observation is that people with low tolerance for cardiovascular risk factors die before they reach old age and that the risk factors associated with metabolic syndrome have less effect on survival in older individuals.

It has been shown that PA may improve the factors that constitute metabolic syndrome [12,25,26]. Therefore, beneficial effects on weight, blood pressure, serum lipid levels and metabolism of carbohydrates may be important for the lower mortality associated with PA among people with metabolic syndrome. Also, improvement in cardiac function, especially in relation to the intensity of PA [27], is likely to play a role.

The question about leisure time PA in the HUNT 2 questionnaire did not distinguish between strength and aerobic exercise, and therefore we cannot attribute the observed effects to a particular type of exercise. People who report being inactive are likely to be inactive; however, PA in questionnaire-based data is typically overreported [28]. In this study, misclassification caused by overreporting of PA would place some people into a higher PA category than their true activity level would indicate. The possible effect of this misclassification would most likely underestimate the effect of PA in this study.

The use of nonfasting glucose values in our study could have been a potential source of bias. Glucose level was used as a diagnostic criterion for metabolic syndrome only if the levels were elevated four hours postprandial. Thus, there was a potential risk of placing some people with metabolic syndrome into the category of people without metabolic syndrome. However, in a subanalysis, glucose was used as a criterion in all subjects regardless of the time since their most recent meal, and the results were essentially the same. Validation of the PA questionnaire in the HUNT 2 study showed that the "light activity" question was not adequately correlated with other measures of PA, whereas the reliability of hard PA and occupational activity was found to be satisfactory [29]. The validation was conducted among 108 men ages 20 to 39 years, however, and therefore we cannot be certain that the results can be generalized to the rest of our study population. In this study, an attempt was made to classify self-reported PA so that it would correspond to the currently recommended guidelines for PA. However, the classification was not exact, and caution should be taken when interpreting these data. Also, some participants might have changed their PA levels after data collection, and these changes might also have influenced the results. However, such changes most likely would have underestimated the differences in mortality between the inactive group and the groups with different activity levels.

## Conclusion

In summary, we found that PA among people with metabolic syndrome was associated with reduced risk of death from all causes and from CVD. The reduction displayed a dose-risk association, but maybe equally importantly, we found that even a low level of PA was associated with a substantial mortality reduction compared to those who reported that they were physically inactive. Our results strongly indicate that PA should be recommended to people with metabolic syndrome to reduce their risk of premature death.

## Competing interests

The authors declare that they have no competing interests.

## Authors' contributions

DS developed the idea of conducting the study, analysed the data, interpreted the results and wrote the paper. JN analysed the data, interpreted the results and wrote the paper. TILN analysed the data and interpreted the results. UW conceived the idea of the study, interpreted the results and wrote the paper. SAS conceived the idea of the study and wrote the paper. LJV interpreted the results and wrote the paper. All authors read and approved the final manuscript.

## Pre-publication history

The pre-publication history for this paper can be accessed here:

http://www.biomedcentral.com/1741-7015/9/109/prepub
